# Outcomes after emergency appendicectomy in patients with liver cirrhosis: a population-based cohort study from England

**DOI:** 10.1007/s00423-023-03072-3

**Published:** 2023-09-18

**Authors:** Alfred Adiamah, Adil Rashid, Colin J. Crooks, John S. Hammond, Peter Jepsen, Joe West, David J. Humes

**Affiliations:** 1 National Institute for Health Research Nottingham Digestive Diseases Biomedical Research Unit, Nottingham University Hospitals NHS Trust, E Floor West Block, QMC Campus, Nottingham, NG7 2UH UK; 2 Division of Hepatobiliary and Transplant Surgery, Freeman Hospital, Freeman Rd, High Heaton, Newcastle Upon Tyne, NE7 7DN UK; 3 Department of Hepatology and Gastroenterology, Aarhus University Hospital, Aarhus, Denmark; 4 Population and Lifesciences, School of Medicine, University of Nottingham, Clinical Sciences Building, City Hospital, Nottingham, NG5 1PB UK

**Keywords:** Cirrhosis, Appendicectomy, Emergency surgery, Postoperative mortality

## Abstract

**Introduction:**

The mortality risk after appendicectomy in patients with liver cirrhosis is predicted to be higher than in the general population given the associated risk of perioperative bleeding, infections and liver decompensation. This population-based cohort study aimed to determine the 90-day mortality risk following emergency appendicectomy in patients with cirrhosis.

**Methods:**

Adult patients undergoing emergency appendicectomy in England between January 2001 and December 2018 were identified from two linked primary and secondary electronic healthcare databases, the clinical practice research datalink and hospital episode statistics data. Length of stay, re-admission, case fatality and the odds ratio of 90-day mortality were calculated for patients with and without cirrhosis, adjusting for age, sex and co-morbidity using logistic regression.

**Results:**

A total of 40,353 patients underwent appendicectomy and of these 75 (0.19%) had cirrhosis. Patients with cirrhosis were more likely to be older (*p* < 0.0001) and have comorbidities (*p* < 0.0001). Proportionally, more patients with cirrhosis underwent an open appendicectomy (76%) compared with 64% of those without cirrhosis (*p* = 0.03). The 90-day case fatality rate was 6.67% in patients with cirrhosis compared with 0.56% in patients without cirrhosis. Patients with cirrhosis had longer hospital length of stay (4 (IQR 3–9) days versus 3 (IQR 2–4) days and higher readmission rates at 90 days (20% vs 11%, *p* = 0.019). Most importantly, their odds of death at 90 days were 3 times higher than patients without cirrhosis, adjusted odds ratio 3.75 (95% CI 1.35–10.49).

**Conclusion:**

Patients with cirrhosis have a threefold increased odds of 90-day mortality after emergency appendicectomy compared to those without cirrhosis.

**Supplementary Information:**

The online version contains supplementary material available at 10.1007/s00423-023-03072-3.

## Introduction

The overall risk of abdominal surgery in patients with cirrhosis is known to be increased, but the risk of mortality after appendicectomy is poorly defined in the currently available literature. Appendicectomy is the most common emergency abdominal surgical procedure in the UK with over 42,000 procedures undertaken each year. In the general population, appendicectomy is considered a safe procedure, with a 30-day mortality rate of 0.244% that increases in those aged over 60 years and in those with perforated appendicitis [1]. But risk estimates for patients with cirrhosis are conflicting with only unadjusted estimates from very small studies reported [2]. A nationwide database study from the USA that included 376 patients with cirrhosis found no difference in in-hospital mortality following appendicectomy, compared to the general population [3]. In contrast, a Danish population-based study that included 69 patients with cirrhosis estimated 30-day mortality in patients with cirrhosis undergoing appendicectomy to be 9% compared to 0.7% in the general population [4]. These ranges would suggest that patients with cirrhosis have either no risk or a large risk in mortality following appendicectomy. Apart from the lack of adjusting for confounders, these studies were further limited by reporting only in-hospital or 30-day mortality. In patients with cirrhosis, 30-day mortality has been shown to underestimate mortality risk in comparison to 90-day mortality [5].

Given the rising incidence of liver cirrhosis [6], it is anticipated that more patients with the condition will present as an acute emergency with appendicitis and may require surgery. Therefore, this population-based cohort study based in England evaluated the 90-day mortality of patients with cirrhosis undergoing emergency appendicectomy compared to those without cirrhosis.

## Methods

The study gained approval from the Independent Scientific Advisory Committee for Medicines and Healthcare products Regulatory Agency approval board (Protocol 19‐193R).

### Data sources

The Clinical Practice Research Datalink (CPRD) is a primary care database containing diagnostic data for approximately 13 million people in the general population in the UK. Patient diagnoses are coded within the CPRD using Read codes.

Hospital Episode Statistics (HES) is a secondary care database that collects a record for each in-hospital patient care episode delivered in England, by the National Health Service. Records are coded using a combination of ICD-10 codes for diagnosis at discharge along with OPCS-4 codes detailing the procedures performed.

Death certificate data from the Office for National Statistics (ONS) was also used by linking anonymized patient identifiers from the CPRD and HES databases.

The CPRD, HES, and ONS databases have previously been described in detail by Humes et al. [7].

### Study participants

The cohort of patients, aged 18 years and above, were identified using OPCS codes for appendicectomy procedures from the CPRD-HES linked dataset between 1st of January 2001 and 31st of December 2018. ICD-10 codes were used to identify patients with appendicitis. Person‐time at risk commenced on the day before surgery allowing deaths that occurred on the same day as the operation to be included in the analysis. Patients were followed up until they died, left a participating general practice, or for 90 days after surgery.

### Exposed cohort

Patients with liver cirrhosis were identified by the presence of diagnosis or procedure codes related to cirrhosis in the CPRD-HES linked dataset at any time point prior to the date of surgery and 90 days subsequently, using a previously validated code list [8]. In the CPRD database, this included the presence of a Read code for cirrhosis, oesophageal varices and/or portal hypertension. In the HES database, this included the presence of ICD-10 and OPCS-code related to cirrhosis, varices or treatment for varices. Ratib et al. [6] demonstrated that more than 90% of patients with a diagnosis of liver cirrhosis in secondary care also have supportive evidence entered in their primary-care records.

### Covariates

Sex was reported as male or female. Age was categorised into five groups: 18–29 years, 30–49 years, 50–59 years, 60–69 years and 70 years or older. Comorbidity was classified using the Charlson comorbidity index [9] into 0, 1 and ≥ 2. The Charlson comorbidity index was determined from a list of comorbidities from the CPRD-HES linked dataset up to the date of surgery. Index of multiple deprivation (IMD2015) was categorized into quintiles from 1–5 (most to least deprived). Procedures were defined as open or laparoscopic using supplementary OPCS codes, and those patients who underwent laparoscopic converted to open procedures analysed with the open group.

### Outcome definition

The primary outcome was 90-day mortality. This was defined from linked ONS deaths registration records and included all deaths occurring on the date of appendicectomy and up to 90 days after. The secondary outcomes were hospital length of stay and re-admissions within 90 days of discharge defined using the HES database.

### Statistical analysis

The basic characteristics of the cirrhotic and non-cirrhotic cohorts were described using frequencies and percentages for categorical variables, with the chi-squared tests used for significance testing. For continuous variables, medians with their associated interquartile ranges (IQR) are presented.

The crude case fatality rate was calculated from the total number of deaths over the total number of patients per category. Univariate and multivariate logistic regression analysis were used to define the odds ratio of 90-day mortality and readmission following appendicectomy in patients with and without cirrhosis controlling for age, sex and comorbidity. All data management and analyses were performed using Stata® version 16 (StataCorp, College Station, TX, USA).

## Results

### Demographics

In total, 40,353 patients underwent an emergency appendicectomy between 2001 and 2018. Of these, 75 (0.19%) patients had a diagnosis of liver cirrhosis. When comparing patients with and without cirrhosis, there was no significant difference by sex (*p* = 0.982) or socioeconomic status (*p* = 0.066). However, patients with cirrhosis were more likely to be older, median age 48 (IQR 32–65) years compared to 31 (IQR 23–44) years in patients without cirrhosis (*p* < 0.0001) and had a higher comorbidity burden (*p* < 0.0001) (Table 1).

**Table 1:** Basic demographics of cirrhotic and non-cirrhotic patients undergoing appendicectomy

	Cirrhosis	Non-cirrhosis	*P*-value
	*n* (%)	*n* (%)	
Sex			
Female	40 (53.33)	21428 (53.20)	0.982
Male	35 (46.67)	18850 (46.80)	
Age (years)			
18–29	15 (20.00)	19111 (47.45)	< 0.0001
30–49	28 (37.33)	13856 (34.40)	
50–59	9 (12.00)	3351 (8.32)	
60–69	11 (14.67)	2080 (5.16)	
≥ 70	12 (16.00)	1880 (4.67)	
Median (IQR)	48 (32–65) years	31 (23–44) years	< 0.0001
Charlson comorbidity			
0	23 (30.67)	28802 (71.51)	< 0.0001
1	17 (22.67)	8512 (21.13)	
2	35 (46.67)	2964 (7.36)	
Deprivation			
1	19 (25.33)	8552 (21.23)	0.066
2	10 (13.3)	8175 (20.30)	
3	9 (12.00)	8448 (20.97)	
4	17 (22.67)	7875 (19.55)	
5	20 (26.67)	7228 (17.94)	
Operative approach			
Laparoscopic	18 (24.00)	14505 (36.01)	0.030
Open	57 (76.00)	25773 (63.99)	
Year blocks			
2001–2003	11 (14.67)	7369 (18.30)	0.286
2004–2006	10 (13.33)	8510 (21.13)	
2007–2009	22 (29.33)	8349 (20.73)	
2010–2012	16 (21.33)	8046 (19.98)	
2013–2015	10 (13.33)	5729 (14.22)	
2016–2018	6 (8.00)	2275 (5.65)	
Length of stay			
Overall LOS, median (IQR)	4 (3–9) days	3 (2–4) days	< 0.0001
LOS* open appendicectomy	5 (3–11) days	3 (2–5) days	< 0.0001
LOS* lap appendicectomy	3 (2–4) days	2 (2–4) days	0.5224

^*^LOS length of stay

### Change in surgical approach

**Fig. 1:**
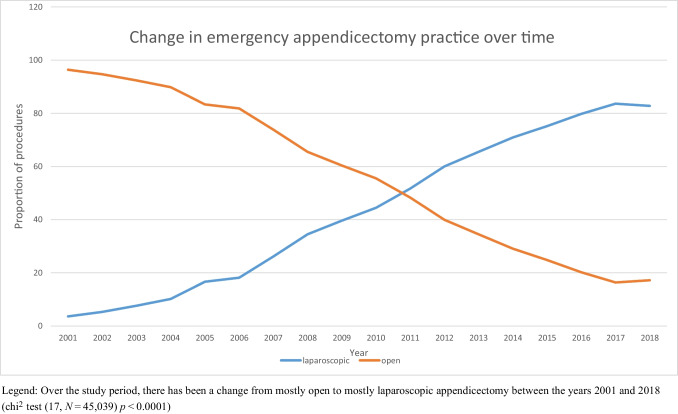
Change in emergency appendicectomy practice in the UK

Overall, 76.00% of patients with cirrhosis underwent an open appendicectomy (OA) compared with 63.99% of patients without cirrhosis (*p* = 0.030) (Table 1). However, from 2001 to 2018, the overall proportion of patients undergoing laparoscopic appendicectomies (LA) increased from 3.62 to 82.80% (*p* < 0.0001) (Fig. 1). This overall trend was mirrored in both patients with (*p* = 0.019) and without cirrhosis (*p* < 0.0001).

### Hospital length of stay

The median hospital length of stay was longer in patients with cirrhosis compared to those without cirrhosis (median 4 (3–9) days vs 3 (2–4) days, *p* < 0.001) (Table 1). The length of stay differed by the approach of surgery with longer length of stay in patients with cirrhosis after open surgery (Table 1).

### Re-admission

In total, 15 (20%) patients with cirrhosis were re-admitted within 90 days of appendicectomy, compared with 4596 (11.41%) of patients without cirrhosis (*p* = 0.019). Readmission was also influenced by the approach to surgery. In those undergoing open appendicectomy, 10.94% (2820/40278) of those without cirrhosis and 21.05% (12/57) of those with cirrhosis were readmitted at 90 days. In univariate analysis, the odds of readmission at 90 days was higher than in non-cirrhotic patients (OR 1.95 (95% CI 1.11–3.43); however, after adjusting for confounders, this was no longer statistically significant (Supplementary Table 1).

### Mortality

The 90-day case fatality following emergency appendicectomy was 0.56% (226/40278) in patients without cirrhosis and 6.67% (5/75) in patients with cirrhosis.

The factors found to be significantly associated with an increased odds of mortality following appendicectomy were cirrhosis, age, comorbidity and operative approach. However, due to significant collinearity between operative approach and cirrhosis, the fully adjusted model accounted for age, sex and co-morbidity (Table 2). It showed that patients with cirrhosis have a threefold increased risk of mortality even after accounting for age, sex and comorbidity, adjusted OR 3.75 (95% CI 1.34–10.49).

**Table 2:** Univariate and multivariate logistical regression of 90-day mortality in patients with and without cirrhosis

	Unadjusted		Adjusted *	
	Odds ratio	95% CI	Odds ratio	95% CI
Cohort				
Non-cirrhotic	1.0	(ref)	1.0	(ref)
Cirrhotic	12.66	5.06–31.66	3.75	1.34–10.49
Sex				
Female	1.0	(ref)	1.0	(ref)
Male	0.90	0.69–1.17	0.83	0.63–1.09
Age (years)				
18–29	1.0	(ref)	1.0	(ref)
30–49	3.03	1.05–8.73	2.93	1.02–8.44
50–59	19.45	7.17–52.75	16.62	6.11–45.17
60–69	76.48	30.19–193.76	54.85	21.48–140.02
≥ 70	346.05	141.863–844.14	202.98	82.02–502.34
No. of co‐morbidities				
0	1.0	(ref)	1.0	(ref)
1	2.06	1.39–3.08	1.40	0.93–2.11
≥ 2	20.04	14.81–27.11	3.22	2.32–4.47

^*^Final model, adjusted for age, sex and co-morbidity

## Discussion

### Findings

Patients with cirrhosis undergoing emergency appendicectomy differed from the patients without cirrhosis, by being older and more comorbid. They had a longer hospital length of stay and had higher rates of readmissions, 20% versus 11%, almost double the readmissions that occurred in patients without cirrhosis. Importantly, the 90-day case fatality following appendicectomy was significantly higher in patients with cirrhosis. Even after adjusting for the differences between the two groups on age, sex and co-morbidity, those patients with cirrhosis had a threefold increased risk of 90-day mortality.

### What is already known

Mortality following appendicectomy in the general population is low. Poulsen et al. [4] estimated 30-day mortality in their general population control to be 0.7% and 9% in those patients with cirrhosis. However, they could not account for any confounding factors. Others have previously noted that both increasing age and comorbidities significantly increase the risk of mortality following appendicectomy [10–12]. Our findings are in line with this in suggesting that both advanced age, co-morbidity and operative approach influence risk of mortality in univariate analysis. Importantly, after adjusting for these confounders, patients with cirrhosis had a threefold increased odds of mortality when compared with patients without cirrhosis.

A nationwide in-patient sample database study from the USA found no difference in in-patient mortality following laparoscopic appendicectomy in 376 patients with cirrhosis and 378 controls [3]. In contrast, a different USA nationwide database study of 438 patients with cirrhosis reported that in-patient mortality following appendicectomy was significantly higher, in comparison to patients without cirrhosis [13]. This difference in studies using similar databases may have arisen from their case definition with the initial study only including patients undergoing laparoscopic appendicectomy. Additionally, the follow-up for mortality was short, restricted to the in-patient stay only. A study from Denmark which had a longer follow-up duration, 30-days, and 69 patients with cirrhosis also found a significantly increased risk of mortality in patients with cirrhosis [4]. However, none of these studies adjusted for confounders, such as age or comorbidities. Our study, with longer follow-up, adjusted for age, sex and comorbidity, has shown an increased odds of mortality following emergency appendicectomy in patients with cirrhosis when compared to those without cirrhosis.

Over the past decade, laparoscopic appendicectomy has become the recommended management of both uncomplicated and complicated appendicitis [14, 15]. Compared to open appendicectomy, laparoscopic appendicectomy has been shown to reduce post-operative mortality, wound infections, postoperative pain and length of hospital stay [12, 16]. One of the earliest consensus statements on laparoscopic surgery suggested cirrhosis was a contraindication. Since then several series in other gastrointestinal surgery have suggested safety of the laparoscopic approach in patients with cirrhosis. In this study, the proportion of patients undergoing laparoscopic appendicectomy increased from 3.6 to 82.8% with a similar trend in uptake in both patients with and without cirrhosis. Suggesting, this is a safe approach.

The results highlight a one day increase in length of stay (LOS) following appendicectomy in patients with cirrhosis. This increase was significant in patients undergoing open appendicectomy and in those who had complicated appendicitis. But in those patients undergoing laparoscopic appendicectomy, there was no difference between those with and without cirrhosis. Previous studies have also demonstrated an increased overall LOS in patients with cirrhosis following appendicectomy [3, 13]. Some of the factors that could explain an increased overall length of stay in the group with cirrhosis include increased risk of acute liver decompensation, fluid and electrolyte imbalance and sepsis, due to reduced immune function in patients with cirrhosis. However, it is important to note that this study did not adjust for confounders, which may contribute to the increased LOS.

### Strengths and limitations

This is the only study to date evaluating 90-day mortality following appendicectomy in patients with and without cirrhosis, adjusted for confounders. However, there are some limitations relevant to all database studies. For example, the dataset relied on accuracy of coding of both the case and exposure definitions. This issue was overcome by only including patients who had both the relevant OPCS codes for appendicectomy and an event date to support that procedure. Additionally, we used a validated algorithm to define cirrhosis in both HES and CPRD data which has been shown to have over 90% concordance when validated against patient notes [17] and defined our outcome of mortality from the ONS data. This provides confidence and reliability in the case definition of appendicectomy, exposure of cirrhosis and outcome of mortality.

This study has shown that the proportion of patients with cirrhosis undergoing appendicectomy has not increased, despite the background rising prevalence of cirrhosis and increasing rates of other procedures in this cohort. This suggests more patients with cirrhosis undergo NOM and outcomes after NOM of appendicitis in patients with cirrhosis needs to be evaluated against outcomes of NOM in patients without cirrhosis, but that was outside the scope of this analysis.

Past studies have demonstrated that operative mortality rises with increasing severity of liver disease [18]. Whilst it would have been possible to subdivide our patients into compensated vs decompensated using the Baveno IV classification, there was insufficient power to do so. We were therefore unable to assess the impact of the underlying severity of cirrhosis (compensated or decompensated) on mortality risk in this analysis.

## Conclusion

Patients with cirrhosis have a threefold increased odds of mortality at 90 days following emergency appendicectomy after accounting for age, sex and co-morbidity. They also have longer hospital length of stay and higher rates of readmission. Careful patient selection, perioperative planning and risk reduction approaches are required to optimise their postoperative outcomes.

### Supplementary Information

Below is the link to the electronic supplementary material.

Supplementary file1 (DOCX 16 KB)

## Data Availability

Data is available on application via the Independent Scientific Advisory Committee for Medicines and Healthcare products Regulatory Agency in the United Kingdom.

## References

[1] Blomqvist PG, Andersson RE, Granath F, Lambe MP, Ekbom AR (2001). Mortality after appendectomy in Sweden, 1987–1996. Ann Surg.

[2] Adiamah A, Ban L, Hammond J, Jepsen P, West J, Humes DJ (2020). Mortality after extrahepatic gastrointestinal and abdominal wall surgery in patients with alcoholic liver disease: a systematic review and meta-analysis. Alcohol Alcohol (Oxford, Oxfordshire).

[3] Al-Azzawi Y, Al-Abboodi Y, Fasullo M, Najuib T (2018). The morbidity and mortality of laparoscopic appendectomy in patients with cirrhosis. Clin Med Insights Gastroenterol.

[4] Poulsen TL, Thulstrup AM, Sorensen HT, Vilstrup H (2000). Appendicectomy and perioperative mortality in patients with liver cirrhosis. Br J Surg.

[5] Neeff HP, Streule GC, Drognitz O, Tittelbach-Helmrich D, Spangenberg H-C, Hopt UT (2014). Early mortality and long-term survival after abdominal surgery in patients with liver cirrhosis. Surgery.

[6] Ratib S, West J, Crooks CJ, Fleming KM (2014). Diagnosis of liver cirrhosis in England, a cohort study, 1998–2009: a comparison with cancer. Am J Gastroenterol.

[7] Humes DJ, Walker AJ, Hunt BJ, Sultan AA, Ludvigsson JF, West J (2016). Risk of symptomatic venous thromboembolism following emergency appendicectomy in adults. Br J Surg.

[8] Fleming KM, Aithal GP, Solaymani-Dodaran M, Card TR, West J (2008). Incidence and prevalence of cirrhosis in the United Kingdom, 1992–2001: a general population-based study. J Hepatol.

[9] Charlson ME, Pompei P, Ales KL, MacKenzie CR (1987). A new method of classifying prognostic comorbidity in longitudinal studies: development and validation. J Chronic Dis.

[10] Andersson MN, Andersson RE (2011). Causes of short-term mortality after appendectomy: a population-based case-controlled study. Ann Surg.

[11] Hui TT, Major KM, Avital I, Hiatt JR, Margulies DR (2002). Outcome of elderly patients with appendicitis: effect of computed tomography and laparoscopy. Arch Surg.

[12] Kotaluoto S, Ukkonen M, Pauniaho SL, Helminen M, Sand J, Rantanen T (2017). Mortality related to appendectomy; a population based analysis over two decades in Finland. World J Surg.

[13] Garcia M, Gerber A, Zakhary B, Finco T, Kazi A, Zhang X (2019). Management and outcomes of acute appendicitis in the presence of cirrhosis: a nationwide analysis. Am Surg.

[14] Di Saverio S, Birindelli A, Kelly MD, Catena F, Weber DG, Sartelli M (2016). WSES Jerusalem guidelines for diagnosis and treatment of acute appendicitis. World J Emerg Surg.

[15] Di Saverio S, Podda M, De Simone B, Ceresoli M, Augustin G, Gori A (2020). Diagnosis and treatment of acute appendicitis: 2020 update of the WSES Jerusalem guidelines. World J Emerg Surg.

[16] Jaschinski T, Mosch CG, Eikermann M, Neugebauer EA, Sauerland S (2018). Laparoscopic versus open surgery for suspected appendicitis. Cochrane Database Syst Rev.

[17] Ratib S, Fleming KM, Crooks CJ, Walker AJ, West J (2015). Causes of death in people with liver cirrhosis in England compared with the general population: a population-based cohort study. Am J Gastroenterol.

[18] Northup PG, Wanamaker RC, Lee VD, Adams RB, Berg CL (2005). Model for end-stage liver disease (MELD) predicts nontransplant surgical mortality in patients with cirrhosis. Ann Surg.

