# Supplementary material for: Outcomes after emergency appendicectomy in patients with liver cirrhosis: a population-based cohort study from England
Source: Langenbecks Arch Surg. 2023 Sep 18;408(1):362. doi: 10.1007/s00423-023-03072-3 (PMC10505594; doi:10.1007/s00423-023-03072-3)
Supplement: Supplementary file 1 — Supplementary file1 (DOCX 16 KB) [file 423_2023_3072_MOESM1_ESM.docx]

Supplementary Table: Univariate and multivariate logistical regression of 90-day readmission in patients with and without cirrhosis

|  | **Unadjusted** | | **Adjusted *** | |
| --- | --- | --- | --- | --- |
|  | **Odds ratio** | **95 % CI** | **Odds ratio** | **95% CI** |
| **Cohort** |  |  |  |  |
| Non-cirrhotic | 1.0 | (ref) | 1.0 | (ref) |
| Cirrhotic | 1.95 | 1.11-3.43 | 1.48 | 0.83-2.62 |
| **Sex** |  |  |  |  |
| Female | 1.0 | (ref) | 1.0 | (ref) |
| Male | 0.80 | 0.75-0.85 | 0.81 | 0.76-0.86 |
| **Age (years)** |  |  |  |  |
| 18-29 | 1.0 | (ref) | 1.0 | (ref) |
| 30-49 | 0.96 | 0.89-1.02 | 0.97 | 0.90-1.03 |
| 50-59 | 0.99 | 0.88-1.11 | 0.94 | 0.84-1.06 |
| 60-69 | 1.15 | 1.01-1.32 | 1.02 | 0.89-1.18 |
| ≥70 | 1.55 | 1.36-1.76 | 1.21 | 1.06-1.40 |
| **No. of co‐morbidities** |  |  |  |  |
| 0 | 1.0 | (ref) | 1.0 | (ref) |
| 1 | 1.42 | 1.32-1.53 | 1.39 | 1.29-1.50 |
| ≥2 | 1.98 | 1.79-2.19 | 1.86 | 1.66-2.08 |

*The final model is adjusted for *“age, sex and co-morbidity”.*
